# Vascular Stem/Progenitor Cells in Vessel Injury and Repair

**DOI:** 10.3389/fcvm.2022.845070

**Published:** 2022-02-10

**Authors:** Jiaping Tao, Xuejie Cao, Baoqi Yu, Aijuan Qu

**Affiliations:** ^1^Department of Physiology and Pathophysiology, School of Basic Medical Sciences, Capital Medical University, Beijing, China; ^2^The Key Laboratory of Cardiovascular Remodeling-Related Diseases, Ministry of Education, Beijing, China; ^3^Beijing Key Laboratory of Metabolic Disorder-Related Cardiovascular Diseases, Beijing, China

**Keywords:** vascular stem cells, atherosclerosis, restenosis, hypertension, aortic aneurysm, vascular injury, vascular remodeling

## Abstract

Vascular repair upon vessel injury is essential for the maintenance of arterial homeostasis and function. Stem/progenitor cells were demonstrated to play a crucial role in regeneration and replenishment of damaged vascular cells during vascular repair. Previous studies revealed that myeloid stem/progenitor cells were the main sources of tissue regeneration after vascular injury. However, accumulating evidences from developing lineage tracing studies indicate that various populations of vessel-resident stem/progenitor cells play specific roles in different process of vessel injury and repair. In response to shear stress, inflammation, or other risk factors-induced vascular injury, these vascular stem/progenitor cells can be activated and consequently differentiate into different types of vascular wall cells to participate in vascular repair. In this review, mechanisms that contribute to stem/progenitor cell differentiation and vascular repair are described. Targeting these mechanisms has potential to improve outcome of diseases that are characterized by vascular injury, such as atherosclerosis, hypertension, restenosis, and aortic aneurysm/dissection. Future studies on potential stem cell-based therapy are also highlighted.

## Introduction

**Figure 1:**
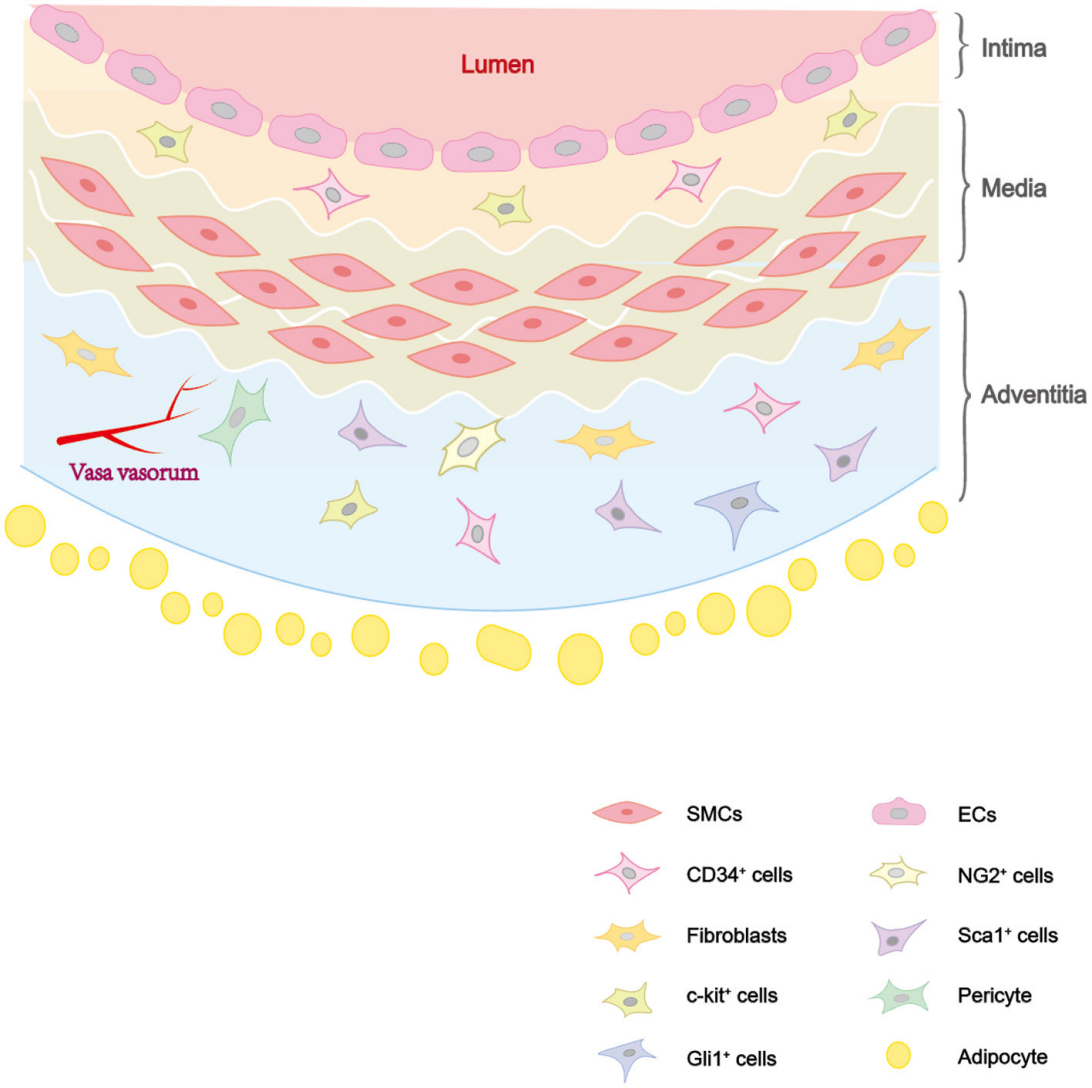
The distribution of cells in vascular wall. The vascular wall consists of three layers: intima, media, and adventitia. The intima consists of ECs and some vascular stem cells such as c-kit^+^ cells. The media is composed of SMCs. The adventitia is composed of vascular stem cells (Sca-1^+^ cells, c-kit^+^ cells, CD34^+^ cells, pericytes, etc.) and fibroblasts.

Vascular injury and repair process has been found to be associated with a variety of cardiovascular diseases, including atherosclerosis, restenosis, hypertension, and arterial aneurysm. They are mainly caused by vascular wall thickening and lumen narrowing. The main causes of the diseases are endothelial injury, vascular smooth muscle cell (VSMC) proliferation, matrix deposition induced abnormal vascular injury and repair (1). The artery wall has three layers as shown in Figure 1: intima, media and adventitia. The intima, which has direct access to blood flow, is mainly made up of endothelial cells (ECs) (2). The media is mainly composed of VSMCs, as well as collagen and elastic fibers. The adventitia is rich in collagen, containing a variety of cells, as well as nerves and blood vessels (3). Previous studies have shown that the occurrence and development of these diseases is due to the phenotypic transformation of VSMCs induced by a variety of factors (4, 5). But there are still unresolved disputations on the reversibility of phenotypic transformation. Recent studies suggested that VSMCs participate in pathological processes, not only through phenotypic transformation, but also from stem/progenitor cells differentiation (6). And it also found that VSMCs derived from stem/progenitor cells participate in atherosclerosis, while maintaining a contractile phenotype without phenotypic transformation (7, 8).

In recent years, studies have demonstrated that resident or circulating stem/progenitor cells, such as endothelial progenitor cells (EPCs), smooth muscle progenitor cells (SMPCs), mesenchymal stem cells (MSCs), and pericytes, can differentiate into several types of vascular cells and form neointima during vascular repair (9). Vascular stem cells (VSCs) residing in blood vessels play a key role in vascular remodeling and closely related to the occurrence of vascular remodeling diseases. During embryonic development, vascular networks depend on vasculogenesis and angiogenesis. Adult VSCs are almost dormant in their niches, once blood vessels are injured, these cells can be activated to initiate neointima, with inflammatory cells infiltration and extracellular matrix (ECM) deposition (10). Perivascular tissues, including the adipose layer is also thought to be crucial in vascular development and disease progression (11).

In this review, we describe several stem/progenitor cells mainly in adventitia and their functions in development of atherosclerosis, restenosis, hypertension, and aortic aneurysm/dissection. At present, there are few studies on VSCs, and the sources of various regenerative ECs and VSMCs involved in the pathological process of vascular remodeling diseases have not been fully studied. Therefore, the mechanism of the participation of VSCs in various vascular remodeling diseases needs to be further studied. In this paper, several types of VSCs and their roles in vascular remodeling-related diseases are reviewed, including various vascular stem/progenitor cells in the process of vascular injury and repair as well as the key elements and important signaling pathways in the occurrence and progression of diseases. New potential targets for clinical treatment of vascular diseases are proposed.

## Vascular Stem/Progenitor Cells in Vessel Wall

During vascular injury, the pathological process of vascular wall changes includes ECs dysfunction, VSMCs proliferation, and inflammatory response. Many studies ‘have demonstrated that vascular stem/progenitor cells can be mobilized in response to various pathological stimuli and play a key role in the vascular repair (12–15). Here we will mainly review studies on EPCs, SMPCs, MSCs, and pericytes (Table 1).

**Table 1:** Distribution and function of vascular wall stem cells in three layers of blood vessels.

**Nomenclature**	**Vessel wall layer**	**Cell markers**	**Function**	**References**
EPCs	Intima	CD31^−^, Flk1^lo^	Differentiate into ECs and participate in angiogenesis	(16)
		CD45^+^, CD14^+^, CD31^+^, CD146^−^, CD133^−^, Tie2^−^	Release VEGF to promote angiogenesis	(17)
	Media and adventitia	CD146^+^, CD45^−^, CD133^−^	Differentiate into ECs	(18, 19)
		PW1^+^	Promote angiogenesis	(20)
		c-kit^+^, VEGFR2^+^, CD45^−^	Differentiate into ECs, VSMCs, and cardiomyocytes	(21, 22)
		CD34^+^, VEGFR2^+^	Differentiate into ECs, hematopoietic cells, and macrophages	(23)
			Prevent the uncontrolled growth of VSMCs	(24–26)
SMPCs	Adventitia	Sca-1^+^, c-kit^+^, CD34^+^, Flk1^+^	Differentiate into VSMCs	(27, 28)
			Differentiate into VSMCs; neointima formation	(29)
	Media and adventitia	Lin^−^ Sca-1^+^ c-kit^−/*lo*^ CD34^−/*lo*^	Differentiate into ECs and VSMCs	(30)
		PW1^+^	Differentiate into VSMCs; promote pulmonary vascular remodeling	(20)
MSCs	Adventitia	CD29^+^, CD44^+^, CD105^−^	Differentiate into osteoblasts, adipocytes, and VSMCs; promote angiogenesis	(31)
Pericytes	Media and adventitia	PDGFRβ^+^	Differentiate into adipocytes, osteoblasts, and chondrocytes;	(32)
		NG2^+^,	Promote vasculogenesis and angiogenesis	(33, 34)
	Media	CD34^+^, CD31^+^, CD45^+^, CD68^+^	Differentiate into myeloid cells, osteoblasts, chondrocytes, and adipocytes	(31)

*EPC, endothelial progenitor cell; SMPC, smooth muscle progenitor cell; MSC, mesenchymal stem cell*.

### Resident Endothelial Progenitor Cells

Earlier studies suggested that EPCs are a group of cells mobilized from bone marrow that participate in endothelium repair after injury (35). In fact, EPCs have a variety of tissue sources, including bone marrow, spleen, blood vessel wall, lipid, and placenta (12). At present, EPCs are defined as the cell population that has the typical clonal proliferation ability and characteristics of stem cells and can differentiate into mature ECs (36).

EPCs actually consist of different cell populations, making up “early EPCs” and “late EPCs” (10). The early stage EPCs appears in early culture stage (4–7 days), its survival time is short and will not differentiate into ECs. EPCs can activate adjacent cells by releasing SDF-1 and VEGF to promote vascular growth (37). It's classical immunophenotypes are CD45^+^,CD14^+^, and CD31^+^ and CD146^−^,CD133^−^, and Tie2^−^ (10). The late stage EPCs can be known as endothelial colony-forming cells (ECFCs), derived from blood vessel wall, human placenta, and white adipose tissue (38). Compared with the early stage EPCs, ECFCs has a higher degree of proliferation and longer survival time, and can differentiate into mature functional ECs to participate in vascular repair (39). It's typical immunophenotypes are CD31^+^, vWF^+^, VE-cadherin^+^, CD146^+^, VEGFR2^+^, and CD45^−^, CD14^−^.

By studying HUVECs and HAECs, it was found that the renewal of vascular wall ECs could be attributed to ECFCs (40). EPCs residing in blood vessels possess multiple abilities such as self-renewal, multi-differentiation potential, and robust proliferation (16). The research pointed out the vital role of Sox18/SoxF transcription factor in the differentiation process. The resident CD157^+^EPCs in vascular wall of large arteries and veins showed regenerative potential of endothelial cells and vasculature (41). Animal studies have proved that EPCs can reduce the formation of neointima after artery injury and restore endothelial function (2). EPCs homing play a central role in vascular remodeling (42). Then neurotrophic factor-3 (NT-3) was proved to be able to accelerate rapid re-endothelialization of damaged carotid arteries by promoting EPCs mobilization and homing (42). It is also regulated by angiogenic chemokines (CXCL1, CXCL7, CXCL12, and CCL2), through their corresponding receptors (CXCR2, CXCR4, and CCR2) (43).

### Smooth Muscle Progenitor Cells

One of the characteristics of SMPCs is the heterogeneity of their origin (44). It is believed that the regenerative VSMCs after vascular injury have several different sources (28). The main functions are contracting blood vessels, regulating angiotasis, and blood pressure (45). There are different sources of VSMCs in different segments of the aorta, expressing different molecular markers and having different functions (2).

Studies have found that if blood vessels are injured, VSMCs will migrate to the intima, changing from a higher differentiated contractile phenotype to a secretory one to initiate cell proliferation (46). It is characterized by increased proliferation, migration, ECM synthesis, along with decreased expression of contraction markers (47). The traditional view holds that imbalance of VSMC plasticity leads to maladaptive phenotypic transformation which leads to the progression of VSMC-driving vascular remodeling diseases (48). But now we found that in vascular injury and repair, sometimes only contractile VSMCs are involved in vascular remodeling process without dedifferentiating to a secretory phenotype (4). Other studies have demonstrated that VSMCs can participate in vascular remodeling process without phenotypic transition (4, 49). From the perspective of stem cell research, recent results have confirmed that VSMCs derived from VSCs can drive vascular remodeling and take part in vascular repair upon injury.

In 2004, Hu et al. (7) first confirmed the presence of Sca-1^+^, c-Kit^+^, CD34^+^, and Flk1^+^ progenitors in the aortic root of *Apoe*^−/−^ mice (36). They found that Sca-1^+^ resident adventitial progenitor cells can migrate and differentiate into VSMCs, playing an important role in atherosclerosis. Later, another group also reported that Sca-1^+^, CD34^+^, and PDGFRβ^+^ cells residented in the adventitia can differentiate into VSMCs *in vitro*. They also reporterd that the process is mediated by acoustic hedgehog (Shh) signaling pathway (50). Shh signaling is limited to the adventitia of the artery and may play a part in the maintenance of VPCs mainly in adventitia (50). Peripheral vascular cells (VSMC and pericytes) may share a common FlK1^+^ progenitor cell with ECs. FlK1 is a key regulator of phenotypic transformation in VSMC which inhibits VSMC differentiation and maintains the Sca-1^+^ progenitor cell phenotype, facilitating multidirectional differentiation and preventing pathological vascular remodeling (47). Based on lineage tracing study, Gli1^+^ cells were also proved to be progenitor cells in the adventitia, expressing CD34, Sca-1, and PDGFRβ and contributing to vascular repair and related diseases (51).

### Mesenchymal Stem Cells

MSCs are pluripotent stromal cells which have proliferative and immunomodulatory effects (52). MSCs from different tissues share the same triadic differentiation potential, that is, the ability to differentiate into osteoblasts, chondrocytes, and adipocytes. The cells both express typical markers CD13, CD73, and CD90 in intima as well as CD29, CD44, and CD105 in adventitia and intima. However, the differentiation tendency of MSCs from different sources also differ (53). Bone marrow-derived MSCs express SH2, SH3, CD29, CD44, CD71, CD90, CD106, CD120a, CD124, and easily differentiate into osteoblasts. Adipose-derived MSCs express CD34, CD13, CD45, CD14, CD144, CD31, and easily differentiate into adipocytes (14, 54). Studies found that adipose tissue stem cells are easier to isolate and more abundant compared with other types of MSCs, they can sidestep ethical issues so are widely used in stem cell studies (55).

Some research groups (56) have isolated a population of CD34^+^CD31^−^ cells expressing pericyte and mesenchymal antigens showing high proliferation and multiple-directional differentiation ability. It can have bidirectional interaction with ECs and take part in angiogenesis. It has been proved that co-culture with ECs on electrostatic spinning scaffolds can promote the differentiation of MSCs into osteoblasts (57). In the mouse model of hind limb ischemia, the researchers also found that EC-like cells derived from MSCs perform strong angiogenesis ability *in vitro* (58). Bone marrow mesenchymal stem cells (BMMSCs) can differentiate into ECs and VSMCs *in vitro* by means of growth factors modulation (59). MSCs are present in the perivascular niche of many organs, including kidneys, lungs, liver, and heart. The vascular-resident Gli1^+^ MSCs are the main cellular source of injury-induced organ fibrosis attributed to their colony-forming activity and differentiation capacity into fibroblasts (60). The effects of these cells in vascular repair depend on their metabolic reprogramming and SMC differentiation ability *via* mir-378a-3p/TGF-β1 signaling pathway (61).

### Pericytes

Pericytes are high density parietal cells located in terminal arterioles and capillaries (62, 63). Pericytes are characterized by direct contact with the underlying ECs. It can regulate capillary permeability, endothelial stability, and micro-vasoconstriction (64). In fact, there are still no specific molecular markers for pericytes. The pericyte markers currently known, such as NG2, CD146, PDGFR-B in media and adventitia, are also expressed in SMPCs and MSCs (64).

In 1992, pericyte was first reported for its osteogenic potential, it has been shown that pericytes reside in capillary and microvessels may be osteoblast progenitors (65, 66). Pericytes and MSCs derived from various human tissues are similar in their function of maintenance vascular homeostasis. Pericytes can help maintain tissue homeostasis by promoting local MSC proliferation and differentiation through the paracrine capability (67). In a systematic investigation, the researchers found the angiogenesis and multilineage differentiation potential of pericytes and MSCs from different human tissues *in vitro*, and enriched CD34^−^CD146^+^ pericytes in adipocytes and bone marrow by magnetic activated cell sorting (15). Only bone marrow-derived cells showed triadic differentiation potential, while adipocyte-derived cells exhibited poor chondrogenic differentiation under TGF-β1 stimulation. These results indicate that the regenerative potential of pericytes as stem cells depends on their tissue origins (15). Recent lineage tracing experiments using the induced Tbx18-CreERT2 cell line showed that pericytes maintained the identity under various pathological conditions such as vascular senescence and will not differentiate into other cells. This discovery challenged the view of pericytes as stem cells (68).

## Vascular Wall Stem/Progenitor Cells in Vascular Diseases

As mentioned above, large amount of vascular stem/progenitor cells participate in the regeneration of damaged ECs and VSMCs during vascular repair upon injury. Numerous studies have confirmed the importance of VPCs in vascular remodeling diseases. Below, the roles of vascular stem/progenitor cells in atherosclerosis, restenosis, hypertension, and aneurysm/dissection, as well as their potential application in regenerative medicine will be reviewed (Table 2).

**Table 2:** Markers and signaling pathways of vascular wall stem cells and related diseases.

**Marker**	**Differentiation**	**Animal models**	**Signaling pathway**	**Related diseases**	**Reference**
c-Kit^+^ CD34^+^	ECs	Aortic root of C-Kit-CreER^T2^; Rosa26-tdTomato mice	AKT/mTOR; Smad2/3	Atherosclerosis; aortic aneurysm	(7, 69, 70)
VEGFR2^+^	ECs	Hind limb ischemia athymic nude mice	GPR4-STAT3/VEGFA	Atherosclerosis; restenosis	(71)
Sca-1^+^	ECs, VSMCs	DKK3^+/+^and DKK3^−/−^; *Apoe* ^−/−^ model; Lepr^+/+^ and Lepr^−/−^ mice	TGF-β/ATF6 and Wnt; OBR-STAT3-MAPK-Rac1/Cdc42-ERK-FAK; Hedgehog	Atherosclerosis; restenosis; hypertension; aortic aneurysm	(7, 50, 72)
CD34^+^, αSMA^+^	ECs, VSMCs	Patients with coronary stenting	Hedgehog; Wnt; Notch	Restenosis	(50, 73)
Pw1^+^	VSMCs	Pw1^nLacZ/+^ mouse	CXCR4; FoxM1	Hypertension	(20, 74)
KLF4	MSCs-like	TGFβR2^iSMC−Apoe^ Mice	TGF-βR2-Smad2/3	Atherosclerosis; aortic aneurysm	(42, 75)
Gli1^+^	MSCs-like	Gli1-CreER^T2^; Ai9 mice	Indian Hedgehog; Smo-Gli12/3; TGFβR2/Smad2/3	Atherosclerosis; hypertension	(51, 60)
EGFP^+^, CD34^+^CD31^−^	SVPs	Human vein	Wnt/β-catenin; Tie-2/PDGF BB; N-cadherin	Aortic aneurysm	(56, 58, 76)
CD34^−^, CD146^+^	PC,MSC-like	Adipocytes and bone marrow	TGF-β1	Atherosclerosis	(56)
NG2^+^	PC,VSMC	Ng2-Cre mice	Ang/Tie2-Calpain/Akt/FOXO3A	Atherosclerosis; aortic aneurysm	(64, 77)

### VPCs in Atherosclerosis

Atherosclerosis is characterized by endothelial damage, VSMC proliferation, and collagen deposition, as well as local thickening of the arterial wall caused by lipid deposition (78). Blood flow in vulnerable areas such as arterial branches has a high oscillation index and a low shear stress (79). This fluid mechanical force can directly act on ECs changing cell morphology, cytoskeleton and intercellular conjunctions, and increasing oxidative stress (80). Low density lipoprotein causes cholesterol storage when attached to the intima of the artery (81). Monocytes enter the intima from the blood, adhere to ECs and turn into macrophage. After phagocytic and ingestion of cholesterol, they form “foam cells” resulting in necrotic nuclei of atherosclerotic plaques with VSMCs (82). Fibrous cap inflammation can determine plaque stability (81). The death of VSMCs, degradation of collagen, and ECM lead to thinning of the vascular cap, resulting in increased plaque rupture with serious consequences. In fact, lumen stenosis often results from repeated ruptures and repair of complex plaques (83).

#### EPCs in Atherosclerosis

**Figure 2:**
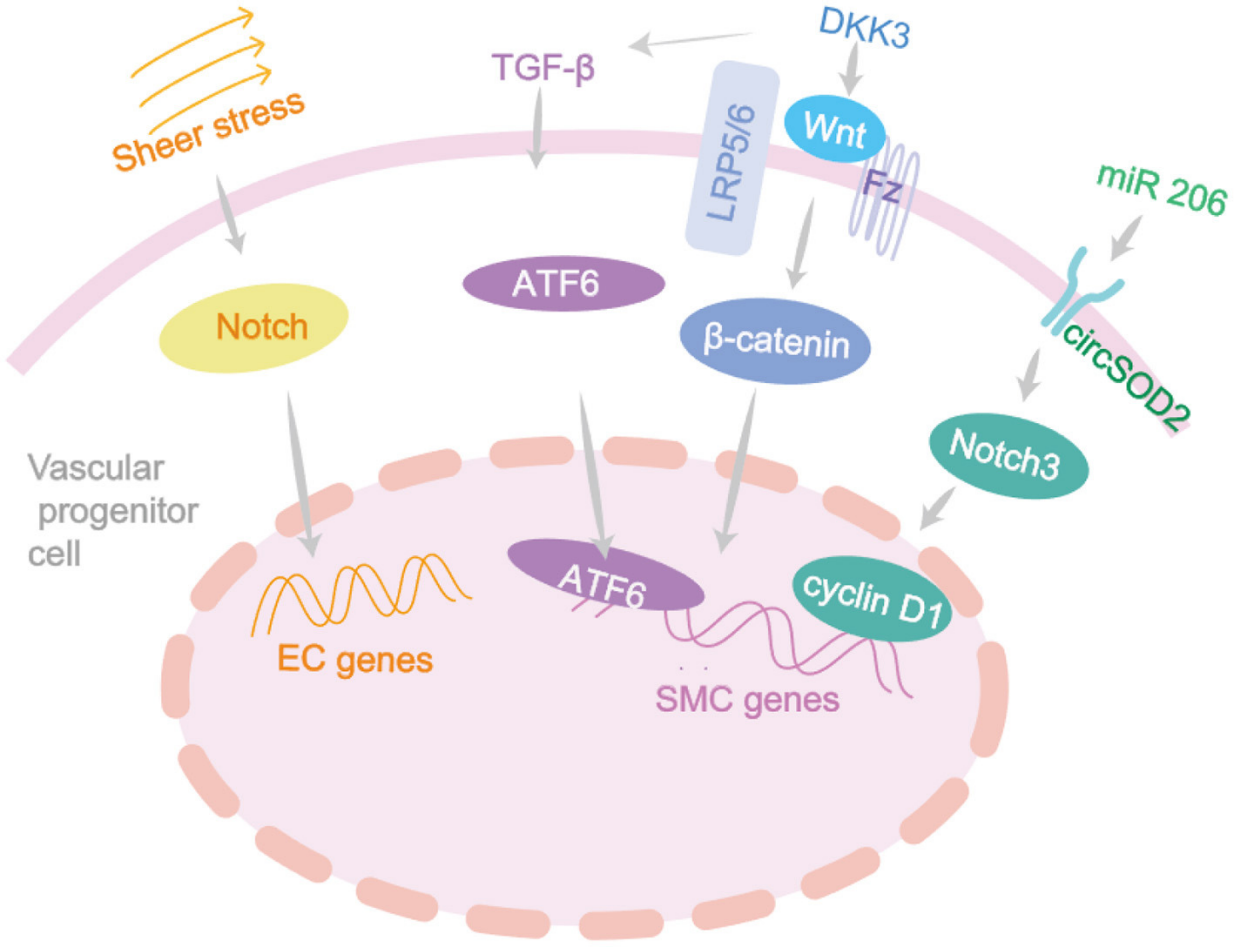
The differentiation signaling pathway of vascular progenitor cells. Vascular development and remodeling depend on many genes and are usually initiated by fluid shear stress which acts on the Notch signaling and closely associated with the vascular remodeling diseases. DKK3 induces ECs migration through Wnt-PCP signaling pathway, accelerating re-endothelialization, and reducing neointima formation. DKK3 can induce the differentiation of Sca-1+ progenitor cells and fibroblasts into VSMC by activating TGF-β/ATF6 signaling pathways.

Decrease in EPCs predicts an increased risk of atherosclerosis. Fluid mechanical forces in arterial branches can directly affect ECs, change cell morphology and increase oxidative stress, inducing renewal of ECs (80). Shear stress is the basic driving factor of EPCs which can promote circulating EPCs homing to the injury site, inducing the anti-atherosclerotic phenotype of EPCs and promoting the differentiation process into mature ECs (84). These effects are mediated by VEGFR2, Tie2, Notch and β 1/3 integrin signaling. In 2020, a new population of EPCs have been identified in vascular walls by single-cell sequencing and lineage tracing techniques (69). The cells can differentiate into luminal and microvascular ECs and participate in endothelial homeostasis in graft sclerosis, with AKT/mTOR dependent glycolysis playing a key role in this process. SDF-1 can mobilize EPCs from bone marrow to the periphery, promote EPC regeneration and prevente cell death under pathological conditions (85). This pathway as mentioned in Figure 2 can provide a potential target for improving the efficacy of progenitor cells in the treatment of vascular diseases (85).

#### SMPCs in Atherosclerosis

In recent years, the roles of SMPCs in cardiovascular diseases have been confirmed in several studies (2, 5). The adventitia progenitor cells expressing Sca-1 markers have been studied in a widespread way. In addition to regulating vascular homeostasis and pathological remodeling, these progenitor cells are also closely related to acute vascular injury in atherosclerosis (86). Initially, the Sca-1^+^ and c-kit^+^ adventitia progenitor cells were found to be capable of differentiating into VSMCs and participate in the formation of venous graft injury. Through the β-Gal-labeled Sca-1^+^ progenitor cells to the adventitia side of vein grafts in *Apoe*^−/−^ mice, the researchers found β-galactose expression in 20% of atherosclerotic lesions (7). Later in 2018, a study implanted GFP-Sca-1^+^ progenitor cells into the adventitia of mice after ligation surgery and found that it could significantly reduce plaque bleeding (87). DKK3 can induce Sca-1^+^ progenitor cells to differentiate into VSMCs by activating the Wnt signaling pathway, and maintain plaque stability (87). It can also induce the differentiation of Sca-1^+^ progenitor cells and fibroblasts into VSMC by activating TGF-β/ATF6 and Wnt signaling pathways (87) (Figure 2).

#### MSCs in Atherosclerosis

During atherosclerosis in *Apoe* intima; these are MSC-like cells expressing Gli1 marker (51). Through acute femoral artery injury repair model, a study confirmed that Gli1^+^MSC-like cells in the adventitia can differentiate into VSMCs. The team also found that Gli1^+^cells can migrate to the media and intima plaques and when calcification occurs in atherosclerosis, Gli1^+^ cells differentiate into osteoblasts (51). In addition, laminar shear stress can activate the Wnt signaling pathway of MSCs, promote β-catenin nuclear transport and activate paracrine factors under laminar shear stress (88).

Many experimental models confirmed the protective effects of MSC therapy on atherosclerosis. The production of Tsg-6, IL-10, NF-κB, and MMP from MSC can inhibit atherosclerotic plaque formation, modulate plaque cellular components and repair endothelial injury, all of which can effectively promote plaque stability (89, 90).

The functions of VPCs in vascular remodeling makes them of great significance in clinical treatment. As EPCs can induce re-endothelialization and angiogenesis of damaged arteries and have a role in regeneration of biological joint structures, they may be a promising clinical therapeutic target (79). Studies have shown a correlation between EPCs dysfunction and reduced angiogenesis in patients with coronary artery disease. As mentioned above, the c-kit^+^ stem/progenitor cells can differentiate into vascular cells to promote post-injury repair. But a recent study shows that most of c-kit^+^ cells differentiate into macrophages and granulocytes to reduce vascular immune inflammatory response to endothelial injury (70). This feature provides a theoretical basis for clinical improvement of vascular disease treatment. *In vitro*, high lipid levels induce MSC migration to intima, initiating the occurrence of atherosclerosis (91). Now MSC transplantation can also be a treatment direction for atherosclerosis (92).

#### VPCs in Restenosis

Percutaneous coronary intervention is an effective treatment for patients with ischemic heart disease. Restenosis is the main complication of percutaneous coronary intervention (93).The main mechanisms are vascular endothelial dissection and subintimal hemorrhage caused by vascular injury after stent implantation (94, 95). Endothelial injury and other irritation can lead to a wound-healing response, while subintimal bleeding can lead to thrombosis and inflammatory cell infiltration (96). Inflammatory factors IL-1, TNF-α, and IL-6 can stimulate the proliferation of VSMCs to form neointima, lead to excessive healing of vascular wall and cause restenosis (97). Neointima is part of the injury repair response, and its formation involves inflammatory cell infiltration, matrix degradation, thrombosis, the proliferation of VSMC, and collagen secretion (98). And the proliferation of VSMCs is the main factor leading to neointima formation and restenosis (99).

The increased secretion of CCL2 and CXCL1 by VSMCs can promote Sca-1^+^ progenitor cell migrate from adventitia to neointima in the injured area. Through the guide wire injury test of mouse femoral artery, the GTPase Rac1/P38 signaling pathway was found to play a key role in this process (100). A study about leptin receptor (OBR) in Sca-1^+^ progenitor cells has found that the OBR-STAT3-MAPK and Rho-GTPase pathways are activated in response to leptin stimulation, inducing progenitor cell migration (72).

**Figure 3:**
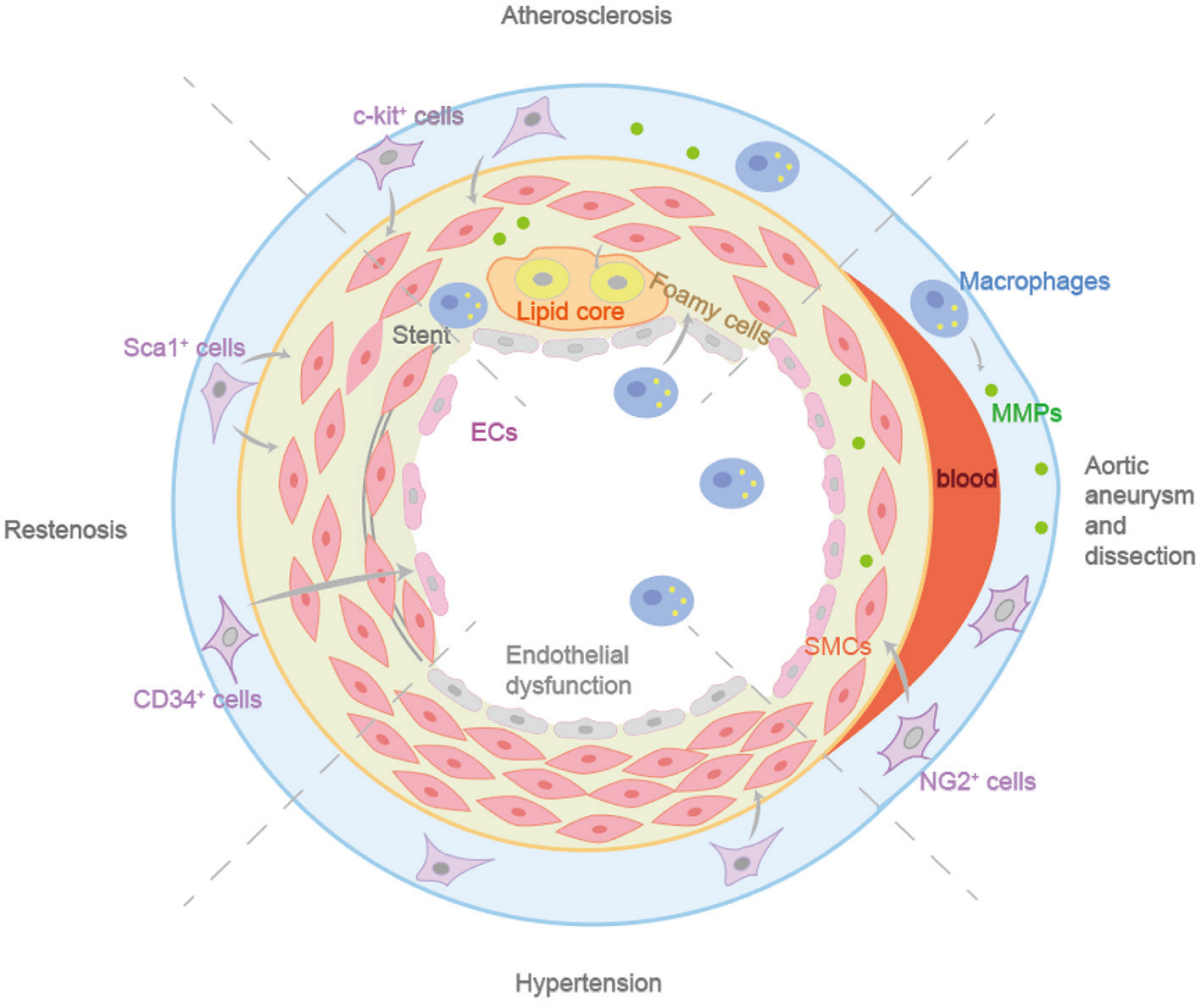
The different roles in vascular remodeling diseases of vascular progenitor cells. c-Kit^+^ cells can differentiate into ECs and participate in endothelial homeostasis. Sca-1^+^ progenitor cells in adventitia can enhance the formation of neointima. Non-bone marrow CD34^+^ cells could differentiate into ECs after femoral artery injury, maintaining vascular integrity and preventing the formation of neointima. Resident NG2^+^ progenitor cells can differentiate into VSMCs after aortic injury during aortic aneurysm and dissection.

The regulatory role of mir-22 in the differentiation of SMPCs has been found, providing a new target for disease treatment of neointimal formation and restenosis (99). A recent study reported that EGFP^+^ MSC in mice expressed endothelial markers and showed the ability of tube formation (58). Meanwhile, these mice showed stronger ability for blood perfusion recovery, vascular density, and improved function of ischemic limbs, providing evidence for its role in angiogenesis. These results suggest that the induction of BMMSCs may be a promising option for the treatment of ischemic diseases (58). Vascular remodeling is known to delay the progression of blood flow restriction during stenosis. However, when restenosis occurs, intimal hyperplasia can lead to lumen narrowing, with adverse effects on the body (101). After bare-metal stent implantation in mice, circulating CD34^+^ cells have a significant increase, which also applies to patients with in-stent restenosis (73). The CD34^+^ cells differentiated into CD31^+^ cells and αSMA^+^ cells after scaffold implantation, which may contribute to the formation of stenosis. Recently, a study found that the elimination of non-bone marrow CD34^+^ cells could reduce the vascular lumen area and increase intima thickness (23). The non-bone marrow CD34^+^ cells could differentiate into ECs after femoral artery injury, maintaining vascular integrity, and preventing the formation of neointima. A regulator CircSOD2, may become a new clinical drug target for inhibiting the development of hyperplastic vascular diseases such as restenosis, because its knockdown can attenuate injury-induced neointima formation and reduce VSMCs proliferation (102). The role of VPCs in vascular remodeling is crucial for improving tissue-engineered vascular grafts and designing drug-targeted therapies for angiogenesis. Dkk3 increases Sca-1^+^ progenitor cell migration and contributes to VSMCs regeneration through CXCR7 activation (103). The DKK3-CXCR7 axis can mediate Sca-1^+^ cell migration and *in vivo* regeneration of transplanted cells, which is of great significance in the study of artificial vascular therapy for restenosis. ETV2-mediated differentiation of exogenous Sca-1^+^ cells into ECs to improve vascular remodeling and reduce neointima after injury (104). Therefore, inducing the differentiation of Sca-1^+^ cells into endothelial lineages might be a therapeutic strategy for vascular diseases as summarized in Figure 3.

### VPCs in Hypertension

Essential hypertension is defined as an unexplained increase in blood pressure that increases the risk of cardiac, brain, or kidney events. Essential hypertension is often associated with hypercholesterolemia, obesity, and diabetes. These risk factors are also present in cardiovascular disease and hypertension can worsen the symptoms (105).The pathogenesis of hypertension includes endothelial dysfunction, sympathetic nervous system activation, inflammation, oxidative stress, etc. Aldosterone can promote the polarization of macrophages to the pro-inflammatory M1 phenotype, leading to vascular dysfunction and aggravating hypertension (106). In hypertension, collagen deposition is the main factor resulting in vascular fibrosis (107). VPCs participate in the occurrence and development of hypertension and vascular fibrosis is related to a variety of signaling pathways.

In the aorta of hypertensive mice induced by Ang II, the number of Sca-1 + progenitor cells increased and the EGFP-Sca-1^+^ progenitor cells co-located with the deposition areas of collagen I, III, and V in the adventitia, suggesting that Sca-1^+^ progenitor cells may be the source of hypertensive vascular fibrosis (76). Lineage tracing technique displayed Adv-Sca-1 cells can differentiate into fibroblasts in the case of vascular injury, leading to vascular remodeling and sclerosis, while KLF4 may maintain the phenotype and prevent pathological vascular remodeling (13). SMPCs express PW1 in the adventitia and participate in pulmonary hypertension-related vascular remodeling (20). The number of PW1^+^ progenitor cells increased significantly in the mouse pulmonary hypertension model under hypoxia and were able to differentiate into VSMCs through the CXCR4 pathway (20). Dysfunctional ECs can induce FoxM1 expression in VSMCs and activate FoxM1-dependent VSMC proliferation, confirming ECs and VSMCs interaction through FoxM1 signaling in vascular remodeling and promoting hypertension and fibrosis (74). Recently, a study induced perivascular fibrosis through Ang II, and found that vascular remodeling during the process was not dependent on blood pressure regulation but through ADAM17 activating PDGFR then acting on ECs (108). ADAM17 may be a new clinical target for preventing hypertension complications (109).

### VPCs in Aortic Aneurysm and Dissection

Aortic aneurysm refers to local dilation of the aortic lumen ≥50% of its original diameter and structural degeneration of different segments of the aorta. Endothelial injury, VSMCs loss and ECM degradation lead to abnormal remodeling of the aortic wall resulting in aortic aneurysm and separation of layers of the aortic wall (aortic dissection) (110, 111). The composition and integrity of ECM are key determinants of the physical properties of the aortic wall (112). ECM undergoes continuous physiological remodeling and the original proteins degraded and replaced by the newly synthesized proteins. MMP plays an important role in this process. Therefore, MMPs are closely related to the pathogenesis of aortic aneurysm (113). Aortic aneurysms can progress gradually, leading to tears in the intima of the aorta or bleeding in the wall of the aorta, further leading to the formation of aortic dissection, which can be life threatening.

Aortic aneurysm formed in two main sites, thoracic aorta and abdominal aorta (114). Abdominal aortic aneurysm (AAA) is most associated with atherosclerosis, they share many common risk factors. Thoracic aortic aneurysms (TAA), located in the aortic root and ascending aorta, are more related with genetic syndromes such as Marfan syndrome (115). AAAs are marked by media and elastic fiber degradation, leading to aortic dissection (116). The pathogenesis of AAA includes VSMCs apoptosis, MMPs degradation of elastin, collagen and glycosaminoglycan, inflammatory reactions and the role of reactive oxygen species (117). Among them, apoptosis of VSMCs and degeneration of aortic media are the hallmark pathological changes of AAA. Studies have found that Il-18 receptor defect, chemokine netrin-1 deficiency, and activated transcription factor EB can reduce apoptosis of VSMCs, decrease the activity of MMP-2/9 and elastin degradation, inhibiting the occurrence and progression of AAA (118). The pathogenesis of TAA includes changes of ECM, apoptosis of VSMCs, MMPs and reactive oxygen species. Mutations of *TGFBR1, TGFBR2, SMAD3, TGFB2, COL3A1, FBN1*, and other genes can cause the occurrence of TAAs (119). For example, firillin-1 is the major subunit of microfibers that constitute the ECM of the thoracic aortic wall. Changes in Firillin-1 result in reduced adhesion between elastic fibers and VSMCs, thereby damaging the biomechanical integrity of the aortic wall and ultimately leading to Marfan syndrome (120). Fibrllin-1 is highly homologous to potential TGFβ binding proteins, and TGFβ gene defects are found not only in Marfan syndrome but also in Patients with Loeys-Dietz aortic aneurysm syndrome (121). A recent discovery found MFAP4 glycosylation was enhanced in MFS patients, and its expression was further enhanced in the advanced aneurysm stage (122). They also induced pluripotent stem cells to differentiate into VSMC in MFS patients and found that there was no elastin expression and the expression level of MFAP4 was unchanged.

VSMC reprogramming to a MSC-like state may play a key role in the progression of AAAs (123). During the pathogenesis of AAA, the TGF-β signaling pathway of VSMCs was inhibited. The decreased expression of TGF-βR2 could lead to reduced binding of Smad2/3 to transcription factor KLF4, leading the transformation of contractile VSMCs into MSCs-like cells. MSCs possess high plasticity and potential to differentiate into several different cell types, such as osteoblasts, chondrocytes, adipocytes, and macrophages. This characteristic has implications for aortic dilation, aortic dilation, calcification of the aortic wall, and inflammation, thereby promoting the development of aneurysms (123). Another research found *Smad3* gene mutations in the second cardiogenic SMPCs and neural crest SMPCs prevented their differentiation into VSMCs and reduced the level of proelastase. This suggests that CPC-SMPCs and NCSC-SMPCs can differentiate into VSMCs through the SMAD3-dependent TGF-β signaling pathway (124). ECs differentiation was reduced in patients with AAAs compared with healthy human MSCs, confirming that the aging MSCs impaired its original vascular repair ability, initiating the occurrence and progress of AAAs (125). Some studies have confirmed that MSCs from calcified and inflammatory aorta have high osteogenic potential and pathological angiogenesis ability under appropriate stimulation (126). Runx2 can enhance the proliferation of MSCs and induce them differentiate into osteoblast lineage cells by regulating Shh, Fgfr2/3, Wnt, and Pthlh signaling pathways (55). Runx2-mediated micro-calcification is a new pathological feature of AAA. It may be a promising strategy for clinical improvement of disease treatment regimens (127). Resident NG2^+^ progenitor cells can differentiate into VSMCs after aortic injury during aortic aneurysm and dissection, producing growth factors that promote endothelial survival and vascular repair (77) (Figure 3).

## Conclusion and Perspective

In this review, we provide the latest research progresses on VSCs in vascular repair upon injury. VSCs exist in the three-layers of vascular wall and play important roles in the occurrence and progression of vascular remodeling diseases such as atherosclerosis, restenosis, hypertension, aortic aneurysm, and dissection. After vascular injury, VSCs can migrate and differentiate into ECs or VSMCs, resulting in the occurrence or aggravation of vascular remodeling-related diseases. Current studies have shown that VSCs can express stem cell markers such as Sca-1 and CD34, but these markers are not specific and their roles in various diseases need to be distinguished from BMSCs or adipose stem cells. The functions of stem cells and their mechanisms in cardiovascular diseases mentioned in the text have not yet been sufficient studied, which is also the direction of further researches. Most groups are now focused on single cell sequencing and cell lineage tracing and other advanced technology to trace the source of all kinds of cells in vascular remodeling associated diseases, in order to better understand the specific role of distinct population of various vascular stem/progenitor cells in vascular remodeling which offers new potential therapeutic targets.

## Author Contributions

BY and AQ conceived and supervised this study. JT, XC, BY, and AQ wrote and revised the manuscript. JT and XC draw the figures and tables. All authors contributed to the article and approved the submitted version.

## Funding

This study was supported by the Key Science and Technology Project of Beijing Municipal Institutions (KZ201910025027 and KZ202010025032); the National Natural Science Foundation of China (81870186 and 82070474); the Importation and Development of High-Caliber Talents Project of Beijing Municipal Institutions (CIT&TCD201904090; CIT&TCD20190332).

## Conflict of Interest

The authors declare that the research was conducted in the absence of any commercial or financial relationships that could be construed as a potential conflict of interest.

## Publisher's Note

All claims expressed in this article are solely those of the authors and do not necessarily represent those of their affiliated organizations, or those of the publisher, the editors and the reviewers. Any product that may be evaluated in this article, or claim that may be made by its manufacturer, is not guaranteed or endorsed by the publisher.

## Abbreviations

AAA: abdominal aortic aneurysm
ADAM17: a disintigrin and metalloproteinase 17
ATF6: activating transcription factor 6
BMMSC: bone marrow mesenchymal stem cells
CCL: Chemokine (C-C motif) ligand
CXCL: Chemokine (C-X-C motif) ligand
CXCR: Chemokine (C-X-C motif) receptor
DKK3: dickkopf 3
EC: endothelial cell
ECFC: endothelial colony-forming cell
ECM: extracellular matrix
EPC: endothelial progenitor cell
Flk-1: fetal liver kinase-1
FoxM1: Forkhead box protein M1
HAECs: human aortic endothelial cells
HUVECs: human umbilical vein endothelial cells
KLF4: krüppel-like factor 4
MACs: myeloid angiogenic cells
MFAP4: microfibrillar-associated protein 4
MFS: marfan syndrome
MMP: matrix metalloproteinase
MSCs: mesenchymal stem cells
NF-κB: nuclear factor-kappa B
PDGF beta: platelets derive growth factors beta
PDGFR: platelet-derived growth factor receptor
SDF-1: stromal cell-derived factor-1
Shh: sonic hedgehog
SMPC: smooth muscle progenitor cell
STAT3: signal transducer and activator of transcription 3
TAA: thoracic aortic aneurysm
TGFβ: transforming growth factor
TNFα: tumor necrosis factor
Tsg6: TNFα-stimulated gene 6
VE-cadherin: vascular endothelial cadherin
VEGF: vascular endothelial growth factor
VSC: vascular progenitor cell
VSMCs: vascular smooth muscle cells
vWF: von willebrand factor.
